# Traumatic Dissection of the Ascending Aorta Complicated by Multiple Injuries Following a Traffic Accident

**DOI:** 10.7759/cureus.74180

**Published:** 2024-11-21

**Authors:** Daigo Shinoda, Kosuke Miyoshi, Nobu Yokoyama, Manabu Shiraishi

**Affiliations:** 1 Department of Cardiovascular Surgery, Tokyo Metropolitan Bokutoh Hospital, Tokyo, JPN

**Keywords:** cardiopulmonary bypass surgery, multiple trauma, optimal surgical timing, traffic accident, traumatic ascending aorta dissection

## Abstract

Trauma to the ascending aorta may progress to a cardiac tamponade, which is often life-threatening. Here, we report on a case of traumatic dissection of the ascending aorta, complicated by multiple injuries. A 24-hour follow-up period was provided to evaluate the traumatic bleeding, and a large tear that extended over three-quarters of the circumference of the aortic intima was identified. An ascending aorta and partial arch replacement were successfully performed. The timing of cardiopulmonary bypass surgery in cases of multiple traumas must be sufficiently flexible to accommodate the condition of each patient, considering the risk of sudden hemodynamic collapse.

## Introduction

Traumatic injuries to the ascending aorta are thought to progress easily to cardiac tamponade, which is often serious at the time of onset and typically not amenable to surgery. Additionally, > 93% of patients with blunt traumatic aortic ruptures die at the site of their accidents [1]. Therefore, survival in such cases is rare. Of the patients who make it to the hospital alive, 75% are initially hemodynamically stable [2]. However, no study has yet determined the optimal timing for surgical intervention in cases of traumatic ascending aortic dissection in patients with multiple injuries.

Traumatic aortic injuries occur predominantly in the aortic isthmus, whereas the ascending aorta is less frequently injured. One theory suggests that hydrostatic pressure rapidly elevates at impact due to deceleration forces, producing a “water hammer” effect, causing the aorta to burst and tear at its weakest point [3]. A prospective study of blunt aortic injury admissions showed that most occurred after head-on collisions (72%) while side impact (24%) and rear impact (4%) collisions occurred less often [2].

Here, we present a rare case of survival following a traumatic dissection of the ascending aorta in a 55-year-old man with multiple injuries.

## Case presentation

A 55-year-old male was brought to the emergency department following traffic-based trauma in which his motor vehicle had struck a median strip in the road. Upon arrival of emergency medical services, the patient reported left anterior chest pain and decreased breath sounds. He was, accordingly, transported to another hospital. Close examination revealed an acute traumatic ascending aortic dissection, pulmonary contusion, traumatic left hemopneumothorax, sternal and rib fractures, zygomatic fracture, right acetabular fracture, left femoral neck fracture, and a cervical spinal cord injury without fracture. As the previous hospital was not equipped for such cases, he was referred to our hospital's emergency center for treatment.

The patient’s vital signs in the emergency department were as follows: Glasgow Coma Scale E4V5M6, blood pressure 120/91 mmHg, pulse rate 109 beats/min, respiratory rate 25 breaths/min, and peripheral oxygen saturation (SpO2) 100% (on a reservoir mask at 10 L/min). As for further neurological findings, no quadriplegia was noted; however, mild upper extremity numbness was observed. No other abnormalities were observed on examination.

Initial blood tests revealed an elevated white blood cell count, creatine kinase, and troponin I while hemoglobin levels were decreased and C-reactive protein was within the normal range (Table 1).

**Table 1:** Blood tests Initial blood tests revealed an elevated white blood cell count, creatine kinase, and troponin I while hemoglobin levels were decreased and C-reactive protein was within the normal range.

	The first blood test at our hospital	Reference range levels
White blood cells	13,700/μL	3100-8400/μL
Hemoglobin	11.7 g/dL	14-18 g/dL
Creatine kinase	981 U/L	59-248 U/L
Troponin I	1817 pg/mL	<4 pg/mL
C-reactive protein	0.06 mg/dL	<0.9 mg/dL

**Figure 1:**
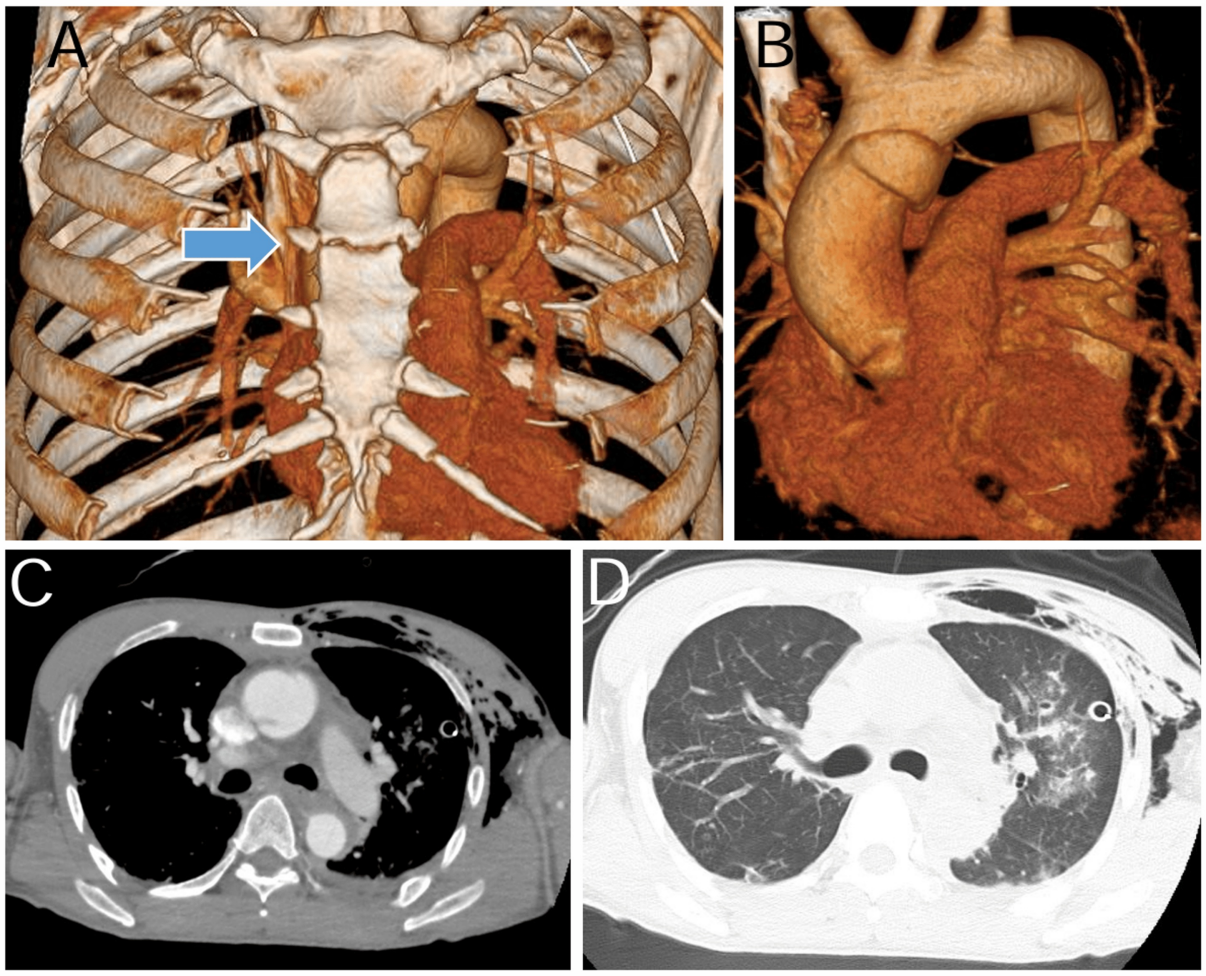
Preoperative contrast-enhanced computed tomography (A) Computed tomography (CT) reveals a sternal fracture; (B) The extent of the aortic dissection is localized with tears from the pulmonary artery to the posterior wall; (C) CT reveals acute ascending aortic dissection and coarse subcutaneous emphysema; (D) CT reveals pulmonary contusion and traumatic left hemopneumothorax Subsequently, a thoracic drain was inserted.

 Contrast-enhanced computed tomography (CT) indicated no increase in pericardial effusion (Figure 1).

In this case, emergency surgery with a cardiopulmonary bypass (CPB) of the multiple traumatic events might have exacerbated his bleeding. In contrast, delayed surgery might have exacerbated the aortic dissection and caused a sudden hemodynamic collapse. We determined that the risk of traumatic hemorrhage related to an emergency aortic replacement with CPB was high, and the patient was monitored closely from the day of admission. He was, therefore, treated conservatively under deep sedation. Hemoglobin levels were consistently maintained above 11 g/dL on serial blood gas analyses performed every six hours until surgery. The following day, laboratory tests confirmed no further progression of anemia or coagulopathy. Physical examination revealed no signs of hematoma, and drainage from the left thoracic drain remained below 10 mL/hour without any increase. Systolic blood pressure was stable within the range of 80-100 mmHg over 24 hours, with no need for additional vasopressor support, and the pulse rate remained steady at 70-80 beats/min, without evidence of tachycardia. Based on these objective parameters, the patient's vital signs were deemed stable, and other injuries were considered controlled. Therefore, surgery was performed.

**Figure 2:**
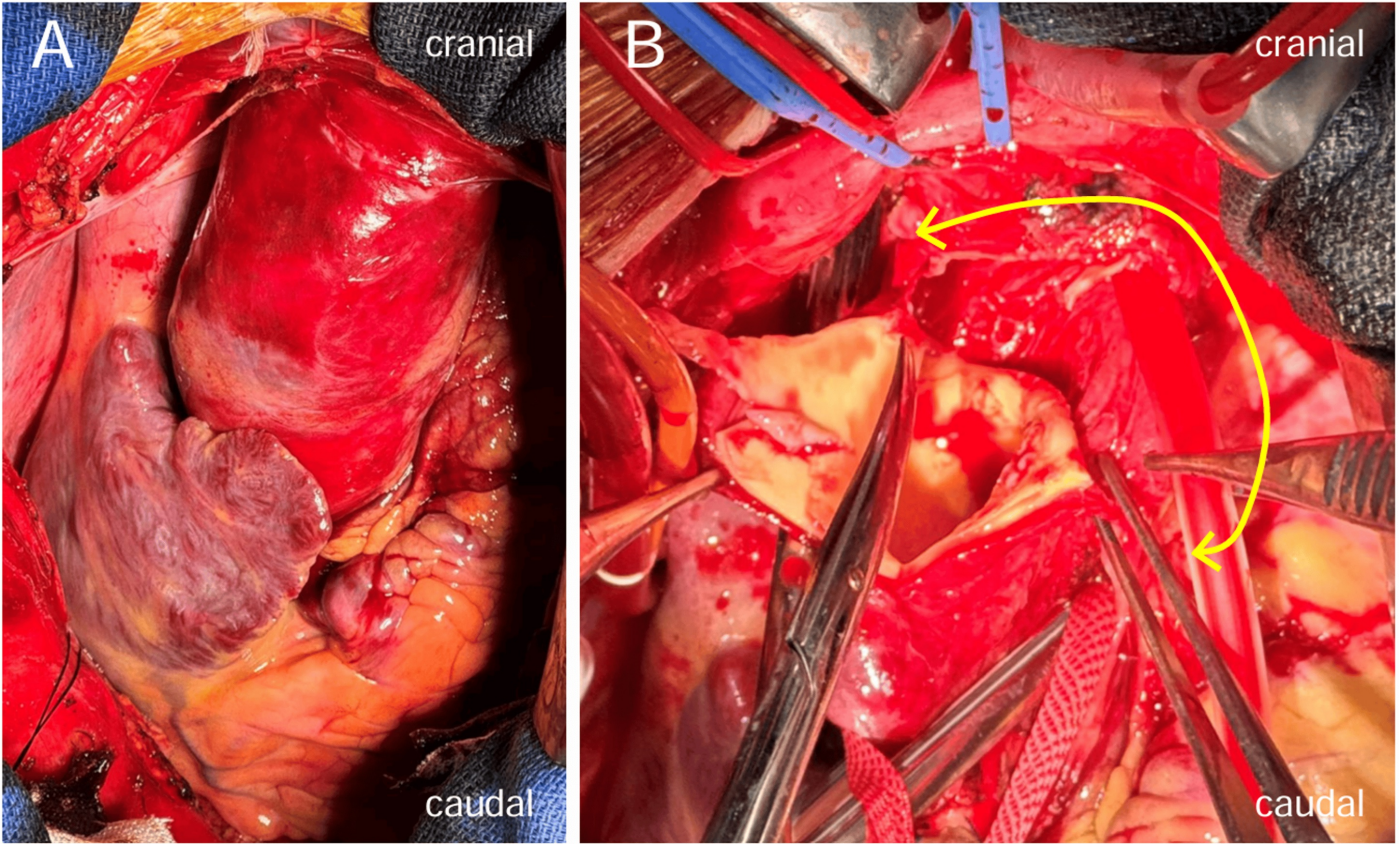
Surgical findings (A) Pericardial incision revealed more than moderate amounts of hemorrhagic pericardial effusion and hematoma on the mediastinal side; (B) The ascending aorta was incised, and a large tear that extended over three-quarters of the circumference of the aortic intima was identified

The surgical approach involved a median sternotomy. Pericardial incision revealed more than moderate amounts of hemorrhagic pericardial effusion and hematoma on the mediastinal side. Heparin was administered at normal doses. CPB was established via cannulation of the right femoral artery, right atrium, and left ventricular vent. After achieving hypothermic circulatory arrest, the ascending aorta was incised, and a large tear that extended over three-quarters of the circumference of the aortic intima was identified (Figure 2).

A small tear was also observed at the orifice of the brachiocephalic artery. A replacement of the ascending aorta and partial arch was performed. CPB weaning was uneventful, and the surgery was completed. Regarding the transfusion strategy, a total of 10 units of red blood cells, 4 units of fresh frozen plasma, and 20 units of platelets were administered according to standard dosing guidelines. More sternal wires were used than usual for sternal fractures to prevent the sternum from shifting. The patient was extubated on postoperative day (POD) 5 due to significant postoperative delirium, which hindered the successful completion of the spontaneous awakening trial. The patient was transferred from the intensive care unit to a general ward on POD 11. No exacerbation of bleeding from other fracture sites was observed, and both neurological impairment and pulmonary injury showed gradual improvement. However, rehabilitation required additional time, and the patient was discharged on POD 83.

## Discussion

Aortic injury can occur in all segments of the aorta. Surgical series demonstrate that 92% of aortic injuries occur at the isthmus with 3%, 4%, and 1% occurring in the ascending aorta, arch, and distal descending aorta, respectively [2]. The ligamentum arteriosum, left main stem bronchus, and paired intercostal arteries limit the movement of the proximal descending aorta. Experiments have suggested that displacement of the aorta in a cranial or caudal direction can cause traction tears at the isthmus [3]. Blunt injury to the thoracic aorta remains the second leading cause of death from vehicular trauma with 94% of victims dying within one hour of injury [2,4]. This case was a traffic-based trauma caused by a car hitting the median strip. As evidenced by the sternal fracture that accompanied the injury, a very high degree of energy was applied to the ascending aorta at the level of the brachiocephalic artery bifurcation, causing dissection. The ascending aorta was shredded three-quarters circumferentially across the posterior aortic wall leading to a moderate hemorrhagic pericardial effusion and a severe life-threatening condition.

Parmley et al. reported that among 275 autopsy cases of traumatic aortic injury, 124 (45%) occurred in the aortic isthmus and 64 (23%) in the ascending aorta. Among these, 47 of 64 (73%) patients with cardiac injury were reported to have an ascending aortic injury. Therefore, ascending aortic injuries are more likely to be complicated by cardiac injuries, suggesting that the fatality rate of ascending aortic injuries is very high [5]. We believe there are three reasons why we were able to save this patient. First, he did not develop a cardiac tamponade instantly, and his circulation did not collapse. Second, no complications were associated with his cardiac injury. Third, no active hemorrhage was associated with the multiple traumas that may have disrupted his cardiovascular dynamics.

Aortic injuries can be treated by open surgery with CPB or by thoracic endovascular aortic repair (TEVAR). Roselli EE et al. reported a case of ascending aortic dissection treated with TEVAR [6]. However this case also showed intimal tears in the brachiocephalic artery, and it is highly possible that the entry closure by TEVAR was technically difficult. In contrast, with traumatic dissection of the ascending aorta involving multiple injuries, determining the optimal timing for surgery with a CPB is challenging. Duwayri et al. reported that a delayed repair is safe and not associated with an increased risk of aortic rupture in hemodynamically stable patients. Delaying aortic surgery in patients with multisystem trauma allows for the optimization of their condition, thereby decreasing perioperative morbidity and mortality [7]. However, no studies have considered the optimal surgical timing for traumatic dissection of the ascending aorta in patients with multiple injuries. In this case, emergency surgery on the same day as the accident had to be avoided because of concerns regarding bleeding complications from multiple traumas associated with the use of CPB and hypothermic circulatory arrest. CT on admission showed no pericardial effusion and no evidence of active aortic bleeding was noted; therefore, the patient was managed with absolute bed rest and strict antihypertensive treatment. In addition, since the patient's circulatory status was stable, no imaging studies were performed immediately before surgery. However, a pericardiotomy revealed a significant amount of blood around the heart during the surgery.

Preoperative management of traumatic ascending aortic dissection needs to be carefully monitored because, as in this case, pericardial effusion can increase at an accelerated rate in a single day. Bellanger et al. stated that delayed cardiac tamponade is a rare phenomenon with two primary causes: delayed hemorrhage after an acute injury or delayed pericarditis with effusion [8]. Even if instantaneous bleeding of the ascending aorta does not result in an accelerated tamponade, it is conceivable that delayed and persistent bleeding from a disruption of the aortic adventitia, which may not be apparent on imaging, may continue and result in a delayed tamponade. In the present case, further delays in surgery were deemed likely to result in sudden hemodynamic collapse. Fortunately, a favorable outcome was achieved. However, flexibility is always important because a uniform surgery waiting period may be risky.

## Conclusions

We successfully treated a patient with an ascending aortic injury due to blunt trauma from a car accident. Preoperative management of traumatic ascending aortic dissection requires careful monitoring, as rapid increases in pericardial effusion can occur within a single day, as observed in this case.

When determining the optimal timing for surgery, it is essential to consider the risk of hemorrhagic complications associated with multiple traumas, as well as the urgency posed by combined aortic and cardiac injuries. A flexible approach is crucial, as adhering to a waiting period of several days may be hazardous.
